# Valerenic Acid and Pinoresinol as Positive Allosteric Modulators: Unlocking the Sleep-Promoting Potential of Valerian Extract Ze 911

**DOI:** 10.3390/molecules30112344

**Published:** 2025-05-27

**Authors:** Roman Senn, Lukas Schertler, Hendrik Bussmann, Juergen Drewe, Georg Boonen, Veronika Butterweck

**Affiliations:** 1 Analytical Department, Max Zeller & Soehne AG, Seeblickstrasse 4, 8590 Romanshorn, Switzerland; roman.senn@zellerag.ch (R.S.); hendrik.bussmann@zellerag.ch (H.B.); 2 Medical Department, Max Zeller & Soehne AG, Seeblickstrasse 4, 8590 Romanshorn, Switzerlandjuergen.drewe@zellerag.ch (J.D.); georg.boonen@zellerag.ch (G.B.)

**Keywords:** *Valeriana officinalis*, valerian, Ze 911, A1A receptor, sleep, positive allosteric modulator, valerenic acid, pinoresinol, VCP 171, CPA

## Abstract

Valerian root extracts are widely used as mild sedatives to promote sleep, with clinical studies confirming their efficacy. Their sleep-promoting effects are associated with the adenosine A1 receptor (A1AR), a key regulator of sleep through neural activity inhibition. Adenosine, a neuromodulator that accumulates during wakefulness, activates A1ARs to facilitate sleep transitions. Using advanced analytics, we detected adenosine at 0.05% in the valerian extract Ze 911, supporting direct A1AR activation in vitro. Additionally, we explored A1ARs’ allosteric sites for modulatory activity. Valerenic acid and pinoresinol, key constituents of Ze 911, were identified as positive allosteric modulators (PAMs) of A1ARs. Valerenic acid exhibited strong PAM activity, with high cooperativity (*αβ* = 4.79 for adenosine and *αβ* = 23.38 for CPA) and intrinsic efficacy (*τB* = 5.98 for adenosine and *τB* = 3.14 for CPA). Pinoresinol displayed weaker PAM activity, with moderate cooperativity (*αβ* = 3.42 for adenosine and *αβ* = 0.79 for CPA) and limited efficacy (*τB* = 0.93 for adenosine and *τB* = 1.66 for CPA). The allosteric modulation observed in valerian extract Ze 911 suggests a mechanism of action in which valerenic acid and pinoresinol enhance receptor activation through allosteric interactions, potentially amplifying the effects of endogenous adenosine. By targeting A1ARs’ allosteric sites, valerian extract Ze 911 offers increased therapeutic selectivity and reduced off-target effects, emphasizing its potential for managing sleep disorders.

## 1. Introduction

Valerian (*Valeriana officinalis* L.; Caprifoliaceae) has long been used as a natural remedy for promoting sleep and relaxation, with its medicinal application dating back to ancient Greece and Rome [1]. Over centuries, valerian root has been utilized to alleviate insomnia, nervousness, and restlessness, and it remains one of the most widely used herbal sleep aids in modern phytotherapy. Despite its well-established use, the precise mechanisms underlying its sedative effects are still being elucidated [2]. Pharmacological studies suggest that valerian may exert its effects through multiple neuromodulatory pathways, including interactions with the adenosine, serotonin, and GABAergic systems [3,4,5,6,7,8].

Valerian roots and rhizomes contain a complex mixture of bioactive compounds such as monoterpenes, sesquiterpenes, flavonoids, caffeic acids, and lignans [9,10,11,12]. Collectively, these compounds contribute to the multifaceted pharmacological effects of valerian, supporting its traditional use in alleviating sleep disorders [1]. Among these bioactive constituents, flavonoids such as hesperidin, and linarin have been recognized for their effects on the central nervous system. Experimental research in mice, using behavioral tests such as thiopental-induced sleeping time, the hole board test, and locomotor activity assessment, demonstrated that both compounds play a significant role in promoting sleep, decreasing exploratory behavior, and reducing overall movement [13]. Of the two, 2S-hesperidin showed the most pronounced sleep-inducing effect, suggesting that it may contribute substantially to valerian’s sedative action [13]. In addition, chlorogenic acid, a compound isolated from *Valeriana officinalis* L., has been shown to exhibit antioxidant activity [14]. Valerenic acid, a prominent sesquiterpene in valerian root, has been identified as a key contributor to the plant’s sedative and anxiolytic effects. It has been shown to positively modulate GABA-induced chloride currents in GABA_A_ receptors, while its derivative, acetoxy valerenic acid, appears to exert an opposite effect [3,5,8,15]. Furthermore, valerenic acid exhibits spasmolytic and muscle relaxant properties, further supporting its use in managing nervous tension and muscle spasms [16].

Studies further indicate that valerian exerts adenosine-like activity by modulating A1AR activity [6,17,18]. Experimental evidence suggests that a specific olivil derivative present in valerian acts as a partial agonist at both rat and human A1 receptors [18]. Additionally, valerian extract Ze 911 has been shown to inhibit post-synaptic potentials in rat cortical neurons in a dose-dependent manner, an effect that could be reversed by A1 receptor antagonists [7,19]. These findings suggest that certain constituents of valerian contribute to its sedative properties by enhancing the sleep-promoting effects of endogenous adenosine. Furthermore, valerian extract Ze 911 has been shown to attenuate the stimulant effects of caffeine in human volunteers, thereby providing further evidence for its interaction with the adenosine system and highlighting the clinical relevance of its agonistic activity at the A1 receptor [20].

Adenosine is a central neuromodulator that accumulates in the brain during prolonged wakefulness and is widely recognized as a fundamental sleep-promoting factor [21,22,23]. It exerts its effects primarily through A1A and A2A receptors, with A1AR activation leading to the inhibition of wake-promoting neurotransmitters such as glutamate, acetylcholine, and orexin [24]. During wakefulness, neuronal activity and ATP breakdown result in a gradual increase in extracellular adenosine levels, which contributes to sleep pressure by reducing neuronal excitability and synaptic transmission. As sleep progresses, adenosine levels decline, allowing wake-promoting systems to regain activity. The importance of adenosine in sleep regulation is further highlighted by the effects of adenosine receptor antagonists such as caffeine, which promote wakefulness by blocking A1A and A2A receptor signaling [23,24]. Given its role in modulating neural activity, the A1AR has been explored as a pharmacological target for sleep disorders [23,24,25]. Traditional A1AR agonists, however, have shown limited clinical success due to poor selectivity, as the orthosteric binding site is highly conserved across adenosine receptor subtypes, leading to off-target effects on A2 and A3 receptors. Additionally, on-target cardiovascular effects arise because the A1AR is widely expressed in the heart and brain [25,26].

To overcome these challenges, positive allosteric modulators (PAMs) have emerged as a promising alternative. PAMs bind to a distinct allosteric site, enhancing the receptor’s response to endogenous adenosine only in regions where it is naturally elevated. This spatial and temporal selectivity minimizes systemic side effects. Since allosteric sites are less conserved than orthosteric sites, PAMs can also achieve greater subtype selectivity, avoiding interactions with A2 or A3 receptors [25,27]. Given the critical role of adenosine in sleep regulation and the potential of valerian compounds to modulate A1ARs, this study aimed to investigate whether specific constituents in valerian extract interact with A1ARs through allosteric mechanisms. Understanding these interactions may provide further insight into the pharmacological basis of valerian’s sedative effects and its potential as a therapeutic agent for sleep disorders. In this study, the most commonly known compounds of valerian, including valerenic acid, hesperidin, various hydroxycinnamic acids, and several lignans, were investigated for their potential modulatory effects on the A1AR.

## 2. Results

### 2.1. Direct Agonist Activity and the Role of Adenosine

**Figure 1:**
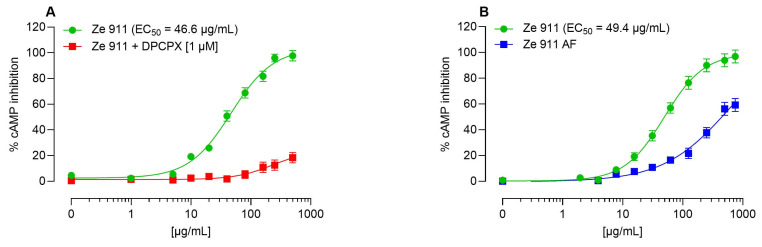
Effects of Ze 911 extract and its adenosine-depleted fraction on cAMP levels in CHO-K1-hA1R cells. CHO-K1 cells stably expressing the human A1 adenosine receptor (hA1R) were co-stimulated with 1 μM forskolin and increasing concentrations of Ze 911 extract or its modified fractions. cAMP levels were measured to assess A1R activation. (**A**) Ze 911 extract alone (green circles) exhibited concentration-dependent inhibition of cAMP formation, with an EC_50_ of 46.6 μg/mL. This effect was effectively blocked by the A1R antagonist DPCPX (1 μM, red squares), confirming that the inhibitory effect is mediated by the A1R via a direct agonist mechanism. (**B**) Comparison of Ze 911 extract (green circles) with Ze 911 AF (adenosine-free extract; blue squares) shows reduced efficacy in the absence of adenosine, indicating that adenosine contributes significantly to the observed A1R activation. Data points represent the mean ± SEM of at least three independent experiments conducted in duplicate. In cases where the SEMs are not visible, they are smaller than the symbol used. These findings support that Ze 911’s agonistic effect on the A1R is primarily driven by its adenosine content.

Ze 911 demonstrated direct activation of the A1AR in cAMP inhibition assays conducted in CHO-K1 cells overexpressing the human A1AR (Figure 1A). This direct effect was antagonized by the selective A1AR antagonist DPCPX, confirming that the observed activity was mediated through the A1AR (Figure 1A).

**Figure 2:**
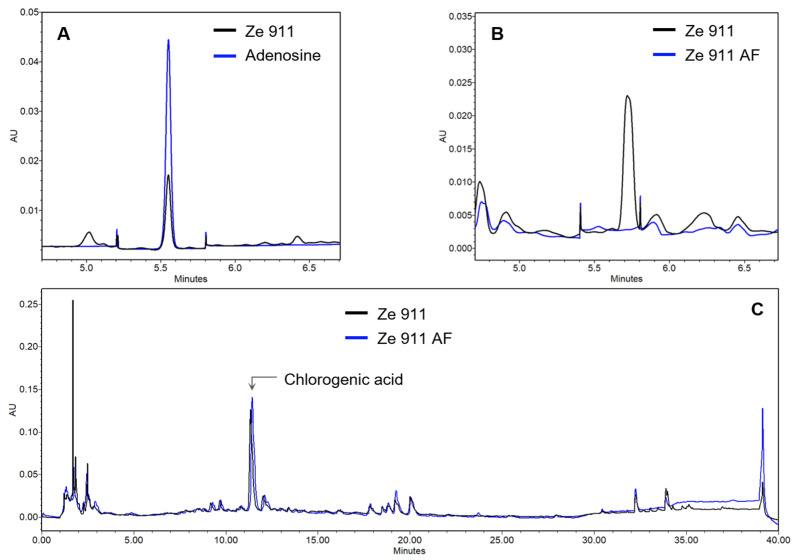
Identification and removal of adenosine from Ze 911 by UPLC. (**A**) Identification of adenosine in Ze 911 by UPLC with PDA detection at 254 nm. (**B**) Overlayed chromatogram demonstrating the absence of the adenosine peak in the Ze 911 AF, confirming the successful removal of adenosine. (**C**) Qualitative comparison of the native and adenosine-free Ze 911 extracts using UPLC-PDA at 254 nm, showing no significant differences in the chromatographic fingerprints.

The adenosine content of Ze 911, quantified at 0.05% using UPLC (Figure 2A), contributed significantly to its direct agonist activity. An adenosine-free extract of Ze 911 was prepared (Figure 2B) to determine the contributions of other active constituents in the extract. An HPLC chromatogram showing the Ze 911 dry extract as well as its adenosine-free version is shown in Figure 2C. The adenosine-free extract of Ze 911 exhibited significantly reduced direct activation of the A1AR, with a rightward shift in the dose–response curve compared to that of the original extract (Figure 1B). This result highlights the role of adenosine as a direct A1AR agonist within Ze 911. The adenosine concentration in Ze 911 at its mean EC_50_ (48 µg/mL) is 90 nM. Adenosine alone has an EC_50_ of 113 nM (Figure S1). Since the extract has a mean EC_50_ of 48 µg/mL, which corresponds to a close but lower adenosine concentration than the effective EC_50_ of pure adenosine, it is suggested that other compounds in the extract contribute synergistically to the observed A1R activation.

### 2.2. Allosteric Modulation of the A1AR

**Figure 3:**
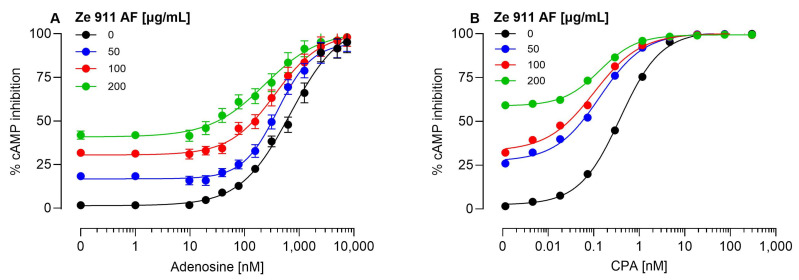
Effect of adenosine-free Ze 911 extract on A1AR agonist-mediated cAMP inhibition in CHO-K1-hA1R cells. CHO-K1 cells stably expressing the human A1 adenosine receptor (hA1R) were co-stimulated with forskolin (1 µM) and increasing concentrations of adenosine (**A**) or CPA (**B**) in the presence of different concentrations of adenosine-free Ze 911 extract (Ze 911 AF). (**A**) Increasing concentrations of Ze 911 AF (50, 100, and 200 µg/mL) progressively enhanced adenosine-induced cAMP inhibition, increasing the maximal response, indicating positive allosteric modulation. (**B**) Similar results were observed with CPA, where Ze 911 AF enhanced CPA-induced cAMP inhibition in a concentration-dependent manner. Data points represent the mean ± SEM of at least three independent experiments conducted in duplicate. In cases where the SEMs are not visible, they are smaller than the symbol used.

Interestingly, the adenosine-free Ze 911 extract demonstrated significant allosteric effects when tested in allosteric mode, suggesting the presence of compounds within the extract that act as PAMs at the A1AR (Figure 3A,B). However, meaningful values for *αβ*, *τB*, and *KB* could not be determined because the extract concentrations were expressed in µg/mL, while the agonist concentrations were provided in molar (M) units.

**Figure 4:**
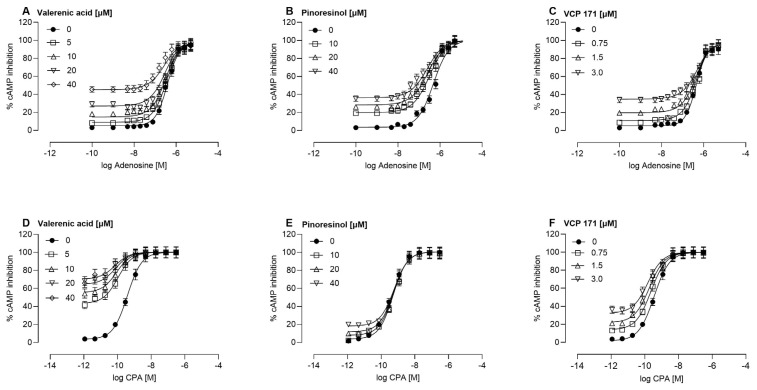
Positive allosteric modulation of adenosine (**A**–**C**) or CPA (**D**,**E**) responses by the test compounds. Effects of valerenic acid (**A**,**D**), pinoresinol (**B**,**E**), or VCP 171 (**C**,**F**) on cAMP inhibition. Each test compound was assessed at various concentrations to evaluate their potential positive allosteric modulation (PAM) properties. Data represent the mean ± SEM of at least three independent experiments conducted in duplicate. In cases where the SEMs are not visible, they are smaller than the symbol used. Curves through the data represent the fit of an operational model of allosterism [28].

For meaningful calculation of these parameters using the operational allosteric ternary complex model [28], both the modulator and agonist concentrations must be expressed in the same units. Despite this limitation, the observed potentiation of the orthosteric agonist response highlights the functional significance of non-adenosine components of the extract. Since the adenosine-free Ze 911 extract demonstrated positive allosteric modulation at the A1AR, further investigations were conducted to identify the active constituents responsible for this effect. Several known valerian-derived compounds, including lignans, sesquiterpenes, flavonoids, and caffeic acid derivatives, were tested for allosteric activity using cAMP inhibition assays. Among the compounds analyzed, only valerenic acid and pinoresinol exhibited significant allosteric modulation, enhancing the efficacy of the orthosteric agonists adenosine (Figure 4C) and N^6^-cyclopentyladenosine (CPA) (Figure 4D–F). No allosteric effects were observed for other tested lignans (Figure S2) or caffeic acid derivatives and selected flavonoids (Figure S3). Valerenic acid exhibited potent positive allosteric modulation at the A1AR (Table 1).

It significantly enhanced the efficacy of the orthosteric agonists adenosine and CPA. The cooperativity values (*αβ*) for valerenic acid were 4.79 and 23.38 for adenosine and CPA, respectively, indicating robust enhancement of agonist binding and receptor activation. Additionally, valerenic acid demonstrated strong intrinsic efficacy, with *τB* values of 5.98 for adenosine and 3.14 for CPA (Table 1). The synthetic PAM VCP 171 showed *αβ* values of 0.37 (adenosine) and 12.73 (CPA), along with *τB* values of 2.63 and 2.00, respectively. Pinoresinol also displayed positive allosteric modulation, albeit with more moderate effects than valerenic acid (Table 1). The cooperativity values for pinoresinol were 3.42 for adenosine and 0.79 for CPA, indicating selective enhancement of adenosine-mediated effects while showing limited potentiation of CPA. The intrinsic efficacy of pinoresinol, reflected in its *τB* values, was lower than that of valerenic acid, with values of 0.93 for adenosine and 1.66 for CPA. These findings suggest that pinoresinol is a weaker modulator overall but retains a degree of selectivity for adenosine as the orthosteric agonist. Valerenic acid exhibited dose-dependent PAM activity at 5, 10, 20, and 40 µM, while pinoresinol was tested at 10, 20, and 40 µM, and VCP 171 at 0.75, 1.5, and 3 µM (Figure 4). Given these differing concentration ranges, direct comparisons between valerenic acid and VCP 171 must be approached cautiously. While valerenic acid demonstrated strong allosteric potentiation, the significantly lower concentrations at which VCP 171 was tested indicate that it may be a more potent PAM on a per-molecule basis. Furthermore, VCP 171 showed PAM activities at relatively low concentrations compared to published data. For example, Cooper et al. [29] could show that VCP 171 at 1, 10, and 30 µM enhanced the binding affinity of orthosteric agonists at both rat and human A1 receptors overexpressed in HEK293T cells, showing clear probe dependence. The study also revealed species-dependent differences in the efficacy of these PAMs, with VCP 171 showing more potent effects in human receptors than in rat receptors. The fact that VCP 171 produced similar effects in HEK293T cells at 3, 10, and 30 µM in the study of Cooper et al. [29] and in CHO cells at 0.75, 1.5, and 3 µM in the present study suggests that differences in receptor expression levels, G-protein coupling efficiency, and cell line sensitivity might contribute to the observed discrepancies in effective concentrations. It is possible that HEK293T cells require higher concentrations to achieve similar effects due to their signaling environment, while CHO cells may display greater sensitivity at lower concentrations. Future studies directly comparing both cell lines under identical assay conditions could help clarify these differences and further establish how cell-dependent factors influence allosteric modulation at A1 receptors.

**Figure 5:**
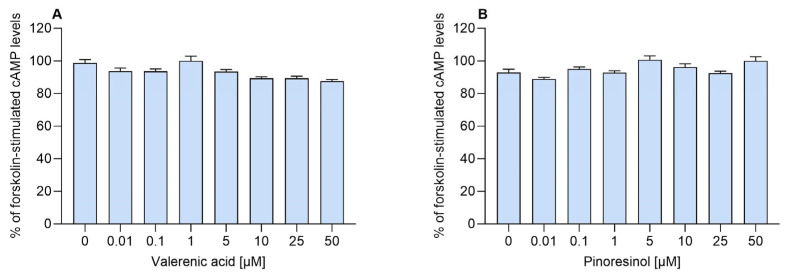
Effects of valerenic acid and pinoresinol on A1AR activity in the absence of adenosine. Effects of different concentrations of (**A**) valerenic acid and (**B**) pinoresinol on forskolin-stimulated cAMP levels in CHO-K1-hA1R cells in the absence of adenosine. Data represent the mean ± SEM of two experiments conducted in triplicate. Neither valerenic acid nor pinoresinol significantly altered forskolin-stimulated cAMP levels across the tested concentrations, indicating no intrinsic agonistic activity on the adenosine A1 receptor in the absence of adenosine.

Finally, to determine whether valerenic acid and pinoresinol exhibit intrinsic agonist activity at the A1AR, both compounds were tested in the absence of adenosine across a concentration range of 0.01 to 50 µM (Figure 5). As shown in Figure 5, no significant reduction in cAMP levels at any tested concentration was observed, indicating that neither compound independently activated the receptor. These findings confirm that valerenic acid and pinoresinol do not function as orthosteric agonists or partial agonists at A1ARs but require the presence of an orthosteric ligand to exert their modulatory effects.

## 3. Discussion

This study provides the first evidence of adenosine in *Valeriana officinalis*, confirming its presence at 0.05% in Ze 911 extract. This finding aligns with previous research that has identified adenosine in plant sources such as tobacco (*Nicotiana tabacum*) and beans (*Phaseolus vulgaris*) [30], highlighting the potential physiological role of plant-derived adenosine. The current data also demonstrate that it is essential to determine the presence of compounds such as adenosine in plant extracts before conducting receptor assays where adenosine acts as an endogenous agonist. Without this analysis, there is a risk of overestimating the effects of tested compounds or misinterpreting receptor activation data. This could lead to inaccurate conclusions about the pharmacological properties of complex extracts, either by masking synergistic effects or falsely attributing activity to other constituents. A comprehensive analysis of plant extracts is therefore crucial to ensure reliable assay outcomes and avoid inaccurate interpretations.

Adenosine is a key physiological molecule involved in nucleic acids, ATP, and cellular signaling. It signals through four GPCRs (A1, A2A, A2B, and A3), influencing neurotransmission, cardiovascular function, inflammation, wound healing, and metabolism [22]. Adenosine accumulates in the brain during wakefulness, acting on A1ARs to reduce neuronal excitability and promote sleep [21,24]. A1ARs play a crucial role in various physiological processes, making them important drug targets. A1AR activation has sedative, anticonvulsant, anxiolytic, and cardioprotective effects, but direct agonists have been limited by side effects such as cardiac disturbances and receptor desensitization [23]. To overcome these issues, strategies such as partial agonists, indirect targeting, and prodrugs have been explored, but allosteric modulation appears most promising. Positive (PAMs) and negative (NAMs) allosteric modulators fine-tune endogenous adenosine activity, offering therapeutic benefits with fewer side effects [27,31]. Unlike orthosteric ligands, allosteric modulators bind distinct receptor sites, enabling selective, physiologically adaptive modulation and reducing tolerance. This approach provides a more precise means of targeting adenosine signaling in disease contexts [26,27,31].

In the present study, Ze 911 was shown to inhibit cAMP signaling in CHO-K1 cells overexpressing the human A1AR, and this effect was fully antagonized by the selective A1AR antagonist DPCPX, confirming receptor-mediated activation. The preparation of an adenosine-free extract allowed for further examination of the contribution of adenosine to the observed effects. This extract exhibited a rightward shift in the dose–response curve, indicating significantly reduced A1AR activation in the absence of adenosine. These results strongly support the conclusion that adenosine is a key orthosteric ligand responsible for A1AR activation in valerian extracts. Although adenosine contributes to A1AR activation, its presence alone does not fully explain the observed pharmacological effects. Calculations of the mean adenosine concentration at the EC_50_ of Ze 911 (48 µg/mL) revealed a value of 90 nM, which is close to, but still below, the EC_50_ of pure adenosine (113 nM). If adenosine were the sole contributor to A1AR activation, the EC_50_ of the extract would be expected to match or be lower than that of pure adenosine. However, the observation that Ze 911 induces receptor activation at adenosine concentrations lower than those required for pure adenosine suggests the involvement of additional active compounds. Several possible explanations could account for this finding. First, other active compounds in Ze 911 may directly contribute to A1AR activation, either by acting as additional agonists or by influencing adenosine metabolism, such as inhibiting adenosine deaminase or nucleoside transporters, thereby prolonging adenosine’s action. Additionally, the combined activity of multiple compounds could result in synergistic effects, where their collective action enhances or sustains A1AR activation beyond what would be expected from adenosine alone. Another possibility is that the extract enhances adenosine’s stability or bioavailability, effectively increasing its concentration at the receptor site.

Beyond its direct agonist effects, non-adenosine compounds in valerian extract Ze 911 also enhance A1AR activity through positive allosteric modulation. Notably, significant allosteric potentiation was observed in a Ze 911–adenosine-free valerian extract, indicating that non-adenosine constituents function as allosteric modulators. Among the valerian-derived compounds tested, valerenic acid and pinoresinol were identified as the key allosteric modulators, significantly enhancing adenosine-mediated responses without directly activating the receptor. This suggests that valerian extract Ze 911 does not solely act by initiating receptor activation but also amplifies endogenous adenosine signaling, thereby increasing the functional efficacy of the receptor in response to physiological adenosine levels. The ability of Ze 911 to enhance endogenous adenosine function through allosteric modulation could contribute to greater selectivity and a lower risk of receptor desensitization than those of direct agonists. Allosteric modulators typically exert their effects only in the presence of the endogenous ligand, preserving the receptor’s physiological regulatory mechanisms while enhancing its natural function. Given these findings, valerian extract Ze 911 represents a distinctive pharmacological tool for modulating A1AR activity. The PAM mode of action may contribute to its therapeutic potential in sleep disorders, anxiety, and neuroprotection, highlighting its relevance in the development of novel adenosine-based therapeutic strategies.

The pharmacokinetic properties of valerenic acid provide additional context for interpreting its role as an A1AR PAM. In human trials, oral administration of 600 mg valerian extract resulted in peak plasma concentrations of valerenic acid ranging from 0.9 to 2.3 ng/mL (~10 nM), with a half-life of approximately 1.1 h [32]. In rats, oral bioavailability was estimated at 33.7%, with an elimination half-life of 2.7–5 h and a high volume of distribution (~17–20 L/kg), suggesting extensive tissue penetration [33]. Maier-Salamon et al. [34] investigated the hepatic metabolism and transport of valerenic acid using isolated perfused livers from Wistar and Mrp2-deficient TR− rats. The data indicate that valerenic acid undergoes extensive hepatic metabolism, primarily through glucuronidation, forming multiple conjugates. The study by Neuhaus et al. [35] investigated the permeability of valerenic acid and its derivatives, hydroxyvalerenic acid and acetoxyvalerenic acid, across an in vitro blood–brain barrier (BBB) model using the ECV304 human cell line. The results showed that all three compounds permeated the BBB significantly slower than diazepam, a known GABA_A_ receptor modulator that crosses via passive transcellular diffusion. The study concluded that valerenic acid and its derivatives do not readily cross the BBB by passive diffusion and likely require an active transport mechanism.

The difference between the plasma concentration of valerenic acid, measured at approximately 10 nM [32], and the significantly higher in vitro concentrations required for positive allosteric modulation of the A1AR, ranging from 10 to 40 µM, does not necessarily imply that the in vitro effects lack relevance in vivo. Several explanations may reconcile the pharmacokinetic data with the observed PAM effects. It is possible that valerenic acid accumulates in specific tissues, including the brain, where its local concentrations could reach pharmacologically relevant levels. Additionally, valerenic acid undergoes extensive metabolic transformation, as shown by Maier-Salamon et al. [34], and some of its metabolites may retain or even enhance its allosteric activity, compensating for its low systemic exposure. Another possibility is that valerenic acid interacts synergistically with other components of Ze 911, leading to an amplified allosteric effect at sub-micromolar concentrations in vivo. In vitro systems are artificial models that may not accurately reflect in vivo pharmacology. Furthermore, cell-based assays often require higher concentrations due to the absence of endogenous adenosine tone, metabolic clearance, and protein interactions. In vivo, A1AR sensitivity differs as receptor activity is influenced by endogenous adenosine, receptor dimerization, and neurotransmitter interactions. Additionally, in vitro studies typically involve acute, high-dose exposure, whereas lower but sustained valerenic acid levels may be sufficient for A1AR modulation in vivo. Moreover, the high concentrations required in vitro may overestimate the dose needed for physiological effects. Since valerenic acid primarily enhances endogenous adenosine signaling rather than directly binding with high affinity, even low levels could significantly potentiate A1AR responses in vivo, particularly when adenosine release is elevated. Thus, physiological conditions absent in cell-based models may amplify its pharmacological effects.

Nguyen et al. [26,36] emphasize the therapeutic advantages of allosteric modulation over traditional orthosteric agonism, particularly in terms of selectivity and safety. Traditional A1AR agonists have been limited by poor selectivity, as the orthosteric binding site is highly conserved among adenosine receptor subtypes, leading to off-target effects on A2 and A3 receptors [37]. Additionally, systemic activation of the A1AR carries risks of on-target cardiovascular suppression due to receptor expression in the heart and vasculature [37]. By contrast, PAMs enhance receptor activity only in the presence of endogenous adenosine, ensuring spatial and temporal selectivity, which reduces systemic side effects [26,27,31]. Furthermore, allosteric sites are less conserved across adenosine receptor subtypes, increasing selectivity for the A1AR and minimizing interactions with A2 or A3 receptors [27]. The ability of PAMs to fine-tune receptor responses in a spatially and temporally controlled manner provides a significant pharmacological advantage, potentially leading to improved therapeutic applications with reduced adverse effects [23,25]. Notably, the identification of valerenic acid as a strong PAM and pinoresinol as a moderate PAM represents a novel finding. While synthetic PAMs such as VCP 171 for adenosine receptors have been well documented, plant-derived PAMs have not been extensively described in the scientific literature [38,39]. Most naturally occurring compounds that interact with adenosine receptors, such as flavonoids and alkaloids, have not been conclusively established as allosteric modulators [40,41,42,43]. The discovery of valerenic acid as a potent natural PAM suggests an unprecedented mechanism of action among plant-derived compounds, positioning valerian as a unique source of allosteric modulation in A1AR signaling. Given that pinoresinol also exhibits moderate PAM activity, this raises the possibility that additional natural PAMs remain undiscovered in botanical sources. The identification of valerenic acid and pinoresinol as A1AR PAMs therefore adds to the growing body of evidence supporting the pharmacological complexity of valerian.

In light of the current findings, future studies should consider exploring the modulatory effects of Ze 911 and its constituents in human neuronal or glial cell lines, or stem cell-derived neural systems, to better simulate native A1AR environments. These models may allow a deeper understanding of receptor signaling, desensitization, and cross-talk in a context more reflective of human physiology. Furthermore, the identification of valerenic acid and pinoresinol as plant-derived PAMs opens new avenues for natural product research. PAMs offer distinct pharmacological advantages—including spatial and temporal selectivity, reduced side effects, and preservation of physiological receptor function—making them attractive for therapeutic modulation in a wide range of conditions, such as anxiety, neurodegeneration, pain, epilepsy, and cardiovascular disorders. The present findings support broader efforts to systematically screen plant-derived compounds for allosteric activity at GPCRs, particularly in the adenosinergic system, where selective modulation remains a critical unmet need. This could pave the way for the development of safer, more targeted therapeutics derived from plant extracts.

## 4. Materials and Methods

### 4.1. Materials

8-Hydroxy-pinoresinol was purchased at Aobious (Glouchester, MA, USA, cat. no. CFN92432). Valerenic acid (cat. no. 89288), (+)-pinoresinol (cat. no. 89525), (−)-olivil (cat. no. 83883), pinoresinol-4-O-Glucoside (cat. no. 84251), chlorogenic acid (cat. no. 89175), pinoresinol-diglucoside (cat. no. 89850), *trans*-ferulic acid (cat. no. 89663), and *trans*-isoferulic acid (cat. no. 89717) were purchased from Phytolab GmbH & Ko KG (Vestenbergsgreuth, Germany). DPCPX (cat. no. C101), coumaric acid (cat. no. C9008), (−)-olivil 4′-*O*-glucoside (cat. no. SMB00261), adenosine (cat. no. A9251), and CPA (cat. no. C8031) were purchased from Sigma-Aldrich (St. Louis, MO, USA). VCP-171 (cat. no. CAY30309) and rolipram (cat. no. AB120029-1002) were purchased from Chemie Brunschwig AG (Basel, Switzerland). Forskolin was purchased from Cisbio (Codolet, France, cat. no. 62AMYADA). Valerian native dry extract (Ze 911, DER native 5—8:1, extraction solvent methanol 50.8% (*v*/*v*), batch number 181111) with a total content of sesquiterpenic acids of 0.41% calculated as valerenic acid was manufactured according to Ph. Eur. EP 9.3/1874 and provided by Max Zeller and Söhne AG, Romanshorn, Switzerland. The adenosine-free extract was generated from a native valerian dry extract (batch number 181111) as described in further detail below.

### 4.2. Cell Culture

*Experiments with adenosine as agonist:* Frozen aliquots of 2.5 × 10^6^ CHO-K1 cells overexpressing the human ADORA1 receptor (PerkinElmer, Rodgau, Germany, cat. no. ES-010-CV) were thawed in a 37 °C water bath and transferred to a 75 cm^2^ culture flask (Corning, Lucerne, Switzerland, cat. no. 430641U) containing 12 mL of Ham’s F12 growth medium (PAN Biotech, Aidenbach, Germany, cat. no. P04-14500, with L-Glutamine and 1.176 g/L NaHCO₃) supplemented with 10% FBS (PAN Biotech, cat. no. P30-193306), 0.4 mg/mL G418 sulfate (Corning, cat. no. 30-234-CI), and 100 U/mL penicillin and 100 µg/mL streptomycin (Corning, cat. no. 30-002-CI). Cells were cultured at 37 °C in a humidified atmosphere with 5% CO_2_ and used for experiments up to passage number 20. At approximately 90% confluence (log-phase), the cells were washed once with 1× PBS (Capricorn Scientific, Ebsdorfergrund, Germany, cat. no. PBS-1A) and detached with 1 mL 0.05% trypsin-EDTA (Gibco Thermo Fisher, Grand Island, NY, USA, cat. no. 25300-062). Cells were diluted to 1 × 10^5^ cells/mL in antibiotic-free growth medium and seeded in triplicates into white 96-well solid-bottom plates (PerkinElmer, Waltham, MA, USA, cat. no. 6005680) at 100 µL per well. The plates were incubated overnight at 37 °C.

*Experiments with CPA as an agonist:* In experiments with CPA as an agonist, inhibition of intracellular cAMP production in response to CPA stimulation of the receptor was determined using a functional assay in CHO-K1 cells overexpressing the human A1 receptor (Eurofins DiscoverX Corporation, Fremont, CA, USA). Cells were seeded in a total volume of 20 μL into white-walled, 384-well microplates and incubated at 37 °C for the appropriate time prior to testing.

### 4.3. cAMP Assay

*Experiments with adenosine as an agonist:* The cAMP assay was initiated after overnight incubation of the plates using the Cisbio cAMP Gi kit (Cisbio, Codolet, France, cat. no. 62AM9PEB/62AM9PEC) with the following modification: Instead of IBMX, the selective phosphodiesterase-4 inhibitor rolipram (Abcam, cat. no. ab120029) was used to prevent cAMP degradation [31]. The kit’s dilution buffer was prepared as a 1× working solution with deionized water, supplemented with 5 µM rolipram, and used to prepare compound treatments and forskolin dilutions. The lysis buffer was used to prepare 1× working solutions of the cryptate-labeled cAMP and anti-cAMP antibody (both supplied as 20x stock solutions). After removing the growth medium, 45 µL of compound treatments at designated concentrations was added to each well and incubated for 45 min. Next, 5 µL of 2 µM forskolin was added to stimulate intracellular cAMP levels, and the plate was incubated for another 45 min. Subsequently, 25 µL of cryptate-labeled cAMP and 25 µL of anti-cAMP antibody were added to each well. Plates were incubated in the dark at room temperature for 1 h. Chemiluminescent signals were measured using a Tecan Spark^®^ Multimode Microplate Reader (Tecan, Männedorf, Switzerland) at 665 nm and 620 nm, as per the manufacturer’s protocol.

A cAMP standard curve was generated in parallel, and the HTRF^®^ Ratio data were plotted against the standard curve to extrapolate cAMP concentrations. The dose–response curve was normalized to the maximal response observed with adenosine (positive control) and the minimal response with vehicle (1% DMSO, negative control). Additionally, cAMP inhibition in response to adenosine was measured in the presence of allosteric modulators.

*Experiments with CPA as an agonist:* cAMP production was measured using the DiscoverX HitHunter cAMP XS+ assay (Eurofins DiscoverX Corporation, Fremont, CA, USA). For allosteric modulation studies, cells were pre-incubated with allosteric modulators at various concentrations, followed by induction with the agonist CPA at its EC_20_ concentration (i.e., the concentration eliciting 20% of the maximal response). Media were aspirated from cells and replaced with 10 μL of HBSS (10 mM HEPES). Sample stocks were diluted to prepare a 4-fold higher (4x) concentration than the final in-well concentration. Next, 5 μL of the 4x compound was added to the cells and incubated at room temperature or 37 °C for 10 min. This was followed by the addition of 5 μL of the 4x EC_20_ agonist solution and further incubation for 30 min at 37 °C. To stimulate intracellular cAMP production and provide a dynamic range for detecting Gi-mediated inhibition, forskolin at its EC_80_ was included in all experiments. The final DMSO (vehicle) concentration was 1%. After incubation with compounds, the assay signal was generated by adding 5 uL of and cAMP XS+ Ab reagent followed by 20 μL of the cAMP XS+ ED/CL lysis cocktail and then incubating for 1 h. This was followed by adding 20 μL of the cAMP XS+ EA reagent and incubating for 2 h at room temperature. Chemiluminescent signals were detected using a PerkinElmer EnVision™ instrument. Compound activity was analyzed using the CBIS data analysis suite (ChemInnovation, San Diego, CA, USA). Control dose–response curves were generated using CPA as the agonist. Data were normalized to the maximal response observed with CPA (positive control) and the minimal response observed with vehicle (1% DMSO, negative control).

### 4.4. Data Analysis

The operational allosteric ternary complex model (ATCM) [28] accounts for the ability of an allosteric ligand to modify affinity and/or efficacy and even activate a receptor independently. This model describes a simplified case for positive allosteric modulators (PAMs), where the orthosteric ligand is a full agonist.

Explanation of parameters: A = Agonist concentration; B = Allosteric modulator concentration; K_B_ = Dissociation constant for the modulator; αβ = Allosteric coupling constant; τB = Intrinsic activity of the modulator; EC_50_ = Half-maximal effective concentration of the agonist; n = Hill coefficient; E_max_ = Maximal system response; Basal = Basal activity (response in the absence of any ligand); Span = E_max_ − Basal (difference between maximum and basal activity).

The response curve Y is defined as follows:

$$Y=Basal+\frac{Span\cdot (Part1)}{\left(Part1+Part2\right)}$$

where

$$Part1={\left(A\cdot \left(K_{B}+\alpha \beta \cdot B\right)+\tau_{B}\cdot B\cdot {EC}_{50}\right)}^{n}$$

$$Part2=({{EC}_{50}}^{n}\cdot {\left(K_{B}+B)\right)}^{n}$$

$$Span=E_{max}-Basal$$

For agonist experiments, curve fitting used non-linear regression analysis applying log(agonist) vs. response data using the following variable slopes:

$$Y=Basal+\frac{\left(Emax-Basal\right)}{\left(1+10^{\left(\left(\log\left({EC}_{50}-X\right)*\;n\right)\right)}\right)}$$

Computerized non-linear regression was performed using Graphpad Prism version 9.0 (Graphpad Software, Boston, MA, USA).

### 4.5. UPLC Analytics

The quantitative measurements of adenosine were carried out using a Waters (Milford, MA, USA) Acquity H-Class UPLC connected to a PDA detector, measuring at a wavelength of 254 nm. An Atlantis dC-18 column (5 µm, 150 × 4.6 mm; Waters) was used, with an injection volume of 7.5 µL (sample dissolved in DMSO). The following gradient was performed: A: water, B: acetonitrile; 0–2 min, 1% B; 2–6 min, 1% to 15% B; 6–6.5 min, 15% to 99% B; 6.5–10 min, 99% B. The flow rate was 1.75 mL/min, and the column temperature was set to 35 °C. A calibration was performed using an adenosine reference standard purchased from Sigma-Aldrich.

An adenosine-free extract was produced using the same chromatographic parameters while coupling the system to an MS detector (Waters QDA) with a programmable switch valve. Within the elution range of adenosine (5.2–5.8 min), the sample flow was directed to MS detection for positive control of adenosine removal while simultaneously excluding the substance from the extract. The collected eluate was dried using nitrogen evaporation. After drying, a small sample amount was analyzed using the same method to qualitatively confirm the absence of adenosine and the integrity of the remaining extract. The qualitative measurements of the extract-fingerprints were carried out on the same UPLC system measuring at a wavelength of 254 nm. A Sunfire C-18 column (3.5 µm, 150 × 3.0 mm; Waters) was used, with an injection volume of 5.0 µL (sample dissolved in DMSO). The following gradient was performed: A: 0.1% formic acid in water, B: 0.1% formic acid in acetonitrile; 0–2 min, 5% B; 2–27 min, 5% to 40% B; 27–32 min, 40% to 99% B; 32–37 min, 99% B. The flow rate was 0.5 mL/min, and the column temperature was set to RT.

## 5. Conclusions

The findings of this study position valerian extract Ze 911 as a unique pharmacological tool for A1AR modulation, with implications for the treatment of sleep disorders and anxiety and for neuroprotection. Beyond direct adenosine-mediated effects, this study identifies valerenic acid as a potent PAM of the A1AR and pinoresinol as a moderate PAM. Given the therapeutic advantages of PAMs over traditional orthosteric agonists, further investigation into valerian’s complex pharmacology could pave the way for novel adenosine-based therapeutic strategies with enhanced efficacy and safety profiles.

## Figures and Tables

**Table 1:** Operational model parameters for the functional allosteric interaction between adenosine or CPA and modulators at the A1AR. Effects of valerenic acid, pinoresinol, and VCP 171 on cAMP inhibition. Analysis was performed according to a simplified operational allosteric ternary complex model [28] in GraphPad Prism. ^a^ Negative logarithm of the equilibrium dissociation constant; ^b^ Antilogarithm of the parameters; ^c^ Logarithm of the operational efficacy parameters of valerenic acid, pinoresinol, and VCP 171 as allosteric agonists; ^d^ Logarithm of the binding (α) and activation (β) cooperativity factors between adenosine or CPA and modulators.

	**Valerenic Acid**		**Pinoresinol**		**VCP 171**	
	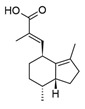		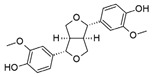		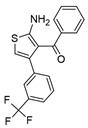	
	**Adenosine**	**CPA**	**Adenosine**	**CPA**	**Adenosine**	**CPA**
**^a^** **logK_B_**	−3.61	−4.75	−4.58	−3.5	−5.02	−4.96
**^b^** **K_B_ [µM]**	243.78	17.42	25.82	316.23	9.38	10.76
**^c^** **LogτB**	0.77	0.49	−0.03	0.22	0.42	0.3
**^b^** **τB**	5.98	3.14	0.93	1.66	2.66	1.99
**^d^** **Logαβ**	0.68	1.36	0.53	−0.1	−0.43	1.11
**^b^** **αβ**	4.79	23.38	3.42	0.79	0.37	12.73
**Goodness of fit (R^2^)**	0.9916	0.9963	0.9921	0.9956	0.9921	0.9961

## Data Availability

The data that support the findings of this study are available from the corresponding author upon reasonable request.

## Supplementary Materials

The following supporting information can be downloaded at: https://www.mdpi.com/article/10.3390/molecules30112344/s1, Figure S1: Dose–response curve of adenosine on cAMP inhibition. Figure S2: Modulation of adenosine A1 receptor (A1AR) activity by lignans. Figure S3: Modulation of adenosine A1 receptor (A1AR) activity by caffeic acid derivatives and flavonoids.

## Abbreviations

The following abbreviations are used in this manuscript:

A1AR	Adenosine A1 receptor
cAMP	Cyclic adenosine monophosphate
CPA	N6-cyclopentyladenosine
GABA	Gamma aminobutyric acid
NAM	Negative allosteric modulator
PAM	Positive allosteric modulator

## References

[1] Bauer R.B.W., Buff W., CLassen B., Heise E.M., Hensel A., Krenn L., Lichius J.J., Lindequist U., Loew D., Melzig M.F., Blaschek W. (2016). Baldrianwurzel—*Valeriana radix*. Wichtl—Teedrogen und Phytopharmaka.

[2] HMPC (2016). Assessment Report on Valeriana officinalis L. Radix and Valeriana officinalis L. Aetheroleum.

[3] Benke D., Barberis A., Kopp S., Altmann K.H., Schubiger M., Vogt K.E., Rudolph U., Mohler H. (2009). GABA A receptors as in vivo substrate for the anxiolytic action of valerenic acid, a major constituent of valerian root extracts. Neuropharmacology.

[4] Dietz B.M., Mahady G.B., Pauli G.F., Farnsworth N.R. (2005). Valerian extract and valerenic acid are partial agonists of the 5-HT5a receptor in vitro. Brain Res. Mol. Brain Res..

[5] Khom S., Strommer B., Ramharter J., Schwarz T., Schwarzer C., Erker T., Ecker G.F., Mulzer J., Hering S. (2010). Valerenic acid derivatives as novel subunit-selective GABAA receptor ligands—In vitro and in vivo characterization. Br. J. Pharmacol..

[6] Muller C.E., Schumacher B., Brattstrom A., Abourashed E.A., Koetter U. (2002). Interactions of valerian extracts and a fixed valerian-hop extract combination with adenosine receptors. Life Sci..

[7] Sichardt K., Vissiennon Z., Koetter U., Brattstrom A., Nieber K. (2007). Modulation of postsynaptic potentials in rat cortical neurons by valerian extracts macerated with different alcohols: Involvement of adenosine A(1)- and GABA(A)-receptors. Phytother. Res..

[8] Trauner G., Khom S., Baburin I., Benedek B., Hering S., Kopp B. (2008). Modulation of GABAA receptors by valerian extracts is related to the content of valerenic acid. Planta Med..

[9] Navarrete A., Avula B., Choi Y.W., Khan I.A. (2006). Chemical fingerprinting of valeriana species: Simultaneous determination of valerenic acids, flavonoids, and phenylpropanoids using liquid chromatography with ultraviolet detection. J. AOAC Int..

[10] Orhan I.E. (2021). A Review Focused on Molecular Mechanisms of Anxiolytic Effect of *Valerina officinalis* L. in Connection with Its Phytochemistry through in vitro/in vivo Studies. Curr. Pharm. Des..

[11] Piccinelli A.L., Arana S., Caceres A., di Villa Bianca R., Sorrentino R., Rastrelli L. (2004). New lignans from the roots of *Valeriana prionophylla* with antioxidative and vasorelaxant activities. J. Nat. Prod..

[12] Wang P.C., Ran X.H., Chen R., Luo H.R., Ma Q.Y., Liu Y.Q., Hu J.M., Huang S.Z., Jiang H.Z., Chen Z.Q. (2011). Sesquiterpenoids and lignans from the roots of *Valeriana officinalis* L. Chem. Biodivers..

[13] Fernandez S.P., Wasowski C., Loscalzo L.M., Granger R.E., Johnston G.A., Paladini A.C., Marder M. (2006). Central nervous system depressant action of flavonoid glycosides. Eur. J. Pharmacol..

[14] Srednicka-Tober D., Hallmann E., Kopczynska K., Goralska-Walczak R., Baranski M., Grycz A., Seidler-Lozykowska K., Rembialkowska E., Kazimierczak R. (2022). Profile of Selected Secondary Metabolites and Antioxidant Activity of Valerian and Lovage Grown in Organic and Low-Input Conventional System. Metabolites.

[15] Hintersteiner J., Haider M., Luger D., Schwarzer C., Reznicek G., Jager W., Khom S., Mihovilovic M.D., Hering S. (2014). Esters of valerenic acid as potential prodrugs. Eur. J. Pharmacol..

[16] Caudal D., Guinobert I., Lafoux A., Bardot V., Cotte C., Ripoche I., Chalard P., Huchet C. (2018). Skeletal muscle relaxant effect of a standardized extract of *Valeriana officinalis* L. after acute administration in mice. J. Tradit. Complement. Med..

[17] Lacher S.K., Mayer R., Sichardt K., Nieber K., Muller C.E. (2007). Interaction of valerian extracts of different polarity with adenosine receptors: Identification of isovaltrate as an inverse agonist at A1 receptors. Biochem. Pharmacol..

[18] Schumacher B., Scholle S., Holzl J., Khudeir N., Hess S., Muller C.E. (2002). Lignans isolated from valerian: Identification and characterization of a new olivil derivative with partial agonistic activity at A(1) adenosine receptors. J. Nat. Prod..

[19] Vissiennon Z., Sichardt K., Koetter U., Brattstrom A., Nieber K. (2006). Valerian extract Ze 911 inhibits postsynaptic potentials by activation of adenosine A1 receptors in rat cortical neurons. Planta Med..

[20] Schellenberg R., Sauer S., Abourashed E.A., Koetter U., Brattstrom A. (2004). The fixed combination of valerian and hops (Ze91019) acts via a central adenosine mechanism. Planta Med..

[21] Bjorness T.E., Kelly C.L., Gao T., Poffenberger V., Greene R.W. (2009). Control and function of the homeostatic sleep response by adenosine A1 receptors. J. Neurosci..

[22] Borea P.A., Gessi S., Merighi S., Vincenzi F., Varani K. (2018). Pharmacology of Adenosine Receptors: The State of the Art. Physiol. Rev..

[23] Pasquini S., Contri C., Merighi S., Gessi S., Borea P.A., Varani K., Vincenzi F. (2022). Adenosine Receptors in Neuropsychiatric Disorders: Fine Regulators of Neurotransmission and Potential Therapeutic Targets. Int. J. Mol. Sci..

[24] Huang L., Zhu W., Li N., Zhang B., Dai W., Li S., Xu H. (2024). Functions and mechanisms of adenosine and its receptors in sleep regulation. Sleep Med..

[25] Pasquini S., Contri C., Cappello M., Borea P.A., Varani K., Vincenzi F. (2022). Update on the recent development of allosteric modulators for adenosine receptors and their therapeutic applications. Front. Pharmacol..

[26] Nguyen A.T.N., Tran Q.L., Baltos J.A., McNeill S.M., Nguyen D.T.N., May L.T. (2023). Small molecule allosteric modulation of the adenosine A(1) receptor. Front. Endocrinol..

[27] Hill S.J., May L.T., Kellam B., Woolard J. (2014). Allosteric interactions at adenosine A(1) and A(3) receptors: New insights into the role of small molecules and receptor dimerization. Br. J. Pharmacol..

[28] Langmead C.J. (2011). Determining allosteric modulator mechanism of action: Integration of radioligand binding and functional assay data. Methods Mol. Biol..

[29] Cooper S.L., Soave M., Jorg M., Scammells P.J., Woolard J., Hill S.J. (2019). Probe dependence of allosteric enhancers on the binding affinity of adenosine A(1)-receptor agonists at rat and human A(1)-receptors measured using NanoBRET. Br. J. Pharmacol..

[30] Johnson L.P., Macleod J.K., Parker C.W., Letham D.S., Hunt N.H. (1981). Identification and quantitation of adenosine-3′:5′-cyclic monophosphate in plants using gas chromatography-mass spectrometry and high-performance liquid chromatography. Planta.

[31] Draper-Joyce C.J., Bhola R., Wang J., Bhattarai A., Nguyen A.T.N., Cowie-Kent I., O’Sullivan K., Chia L.Y., Venugopal H., Valant C. (2021). Positive allosteric mechanisms of adenosine A(1) receptor-mediated analgesia. Nature.

[32] Anderson G.D., Elmer G.W., Kantor E.D., Templeton I.E., Vitiello M.V. (2005). Pharmacokinetics of valerenic acid after administration of valerian in healthy subjects. Phytother. Res..

[33] Sampath C., Haug K., Thanei S., Hamburger M., Derendorf H., Frye R., Butterweck V. (2012). Pharmacokinetics of valerenic acid in rats after intravenous and oral administrations. Planta Med..

[34] Maier-Salamon A., Trauner G., Hiltscher R., Reznicek G., Kopp B., Thalhammer T., Jager W. (2009). Hepatic metabolism and biliary excretion of valerenic acid in isolated perfused rat livers: Role of Mrp2 (Abcc2). J. Pharm. Sci..

[35] Neuhaus W., Trauner G., Gruber D., Oelzant S., Klepal W., Kopp B., Noe C.R. (2008). Transport of a GABAA receptor modulator and its derivatives from *Valeriana officinalis* L. s. l. across an in vitro cell culture model of the blood-brain barrier. Planta Med..

[36] Nguyen A.T., Vecchio E.A., Thomas T., Nguyen T.D., Aurelio L., Scammells P.J., White P.J., Sexton P.M., Gregory K.J., May L.T. (2016). Role of the Second Extracellular Loop of the Adenosine A1 Receptor on Allosteric Modulator Binding, Signaling, and Cooperativity. Mol. Pharmacol..

[37] Kimatrai-Salvador M., Baraldi P.G., Romagnoli R. (2013). Allosteric modulation of A1-adenosine receptor: A review. Drug Discov. Today Technol..

[38] Gao Z.-G., Tosh D.K., Jain S., Yu J., Suresh R.R., Jacobson K.A., Borea P.A., Varani K., Gessi S., Merighi S., Vincenzi F. (2018). A1 Adenosine Receptor Agonists, Antagonists, and Allosteric Modulators. The Adenosine Receptors.

[39] Jacobson K.A., Reitman M.L. (2020). Adenosine-Related Mechanisms in Non-Adenosine Receptor Drugs. Cells.

[40] Yuliana N.D., Khatib A., Link-Struensee A.M., Ijzerman A.P., Rungkat-Zakaria F., Choi Y.H., Verpoorte R. (2009). Adenosine A1 receptor binding activity of methoxy flavonoids from Orthosiphon stamineus. Planta Med..

[41] Muller C.E., Jacobson K.A. (2011). Xanthines as adenosine receptor antagonists. Methylxanthines.

[42] Stolz E.D., da Costa P.F., Medeiros L.F., Souza A., Battastini A.M., von Poser G.L., Bonan C., Torres I.L., Rates S.M. (2016). Uliginosin B, a Possible New Analgesic Drug, Acts by Modulating the Adenosinergic System. Evid. Based Complement. Altern. Med..

[43] Wang M.L., Yu G., Yi S.P., Zhang F.Y., Wang Z.T., Huang B., Su R.B., Jia Y.X., Gong Z.H. (2015). Antinociceptive effects of incarvillateine, a monoterpene alkaloid from Incarvillea sinensis, and possible involvement of the adenosine system. Sci. Rep..

