# Supplementary material for: Valerenic Acid and Pinoresinol as Positive Allosteric Modulators: Unlocking the Sleep-Promoting Potential of Valerian Extract Ze 911
Source: Molecules. 2025 May 27;30(11):2344. doi: 10.3390/molecules30112344 (PMC12155840; doi:10.3390/molecules30112344)
Supplement: Supplementary file 1 [file molecules-30-02344-s001.zip › molecules-3608941-supplementary.pdf]

Article

Valerenic Acid and Pinoresinol as Positive Allosteric Modulators: Unlocking the Sleep-Promoting Potential of Valerian Extract Ze 911

Roman Senn [1,†], Lukas Schertler [2,†], Hendrik Bussmann [1], Jürgen Drewe [2], Georg Boonen [2], Veronika Butterweck [2,*]

1 Analytical Department, Max Zeller & Soehne AG, Seeblickstrasse 4, 8590 Romanshorn, Switzerland; roman.senn@zellerag.ch (R.S.); hendrik.bussmann@zellerag.ch (H.B.)
2 Medical Department, Max Zeller & Soehne AG, Seeblickstrasse 4, 8590 Romanshorn, Switzerland; juergen.drewe@zellerag.ch (J.D.); georg.boonen@zellerag.ch (G.B.)
* Correspondence: veronika.butterweck@zellerag.ch
† These authors contributed equally to this work.

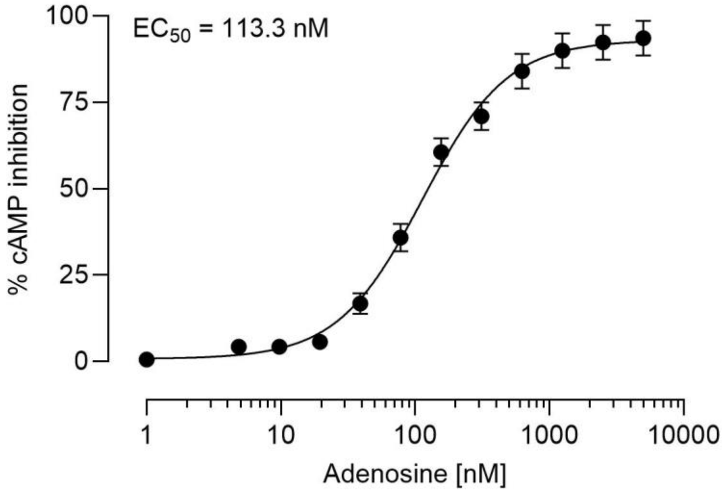

Figure S1. *Dose-response curve of adenosine on cAMP inhibition. The graph shows the concentration-dependent effect of adenosine on cAMP inhibition in in CHO-K1-hA1R cells . Data points represent the mean ± SD of three independent experiments conducted in duplicate. The curve was fitted using nonlinear regression with a sigmoidal dose-response model (Y=Bottom + (Top-Bottom)/(1+10^((LogEC50-X)*HillSlope)).*

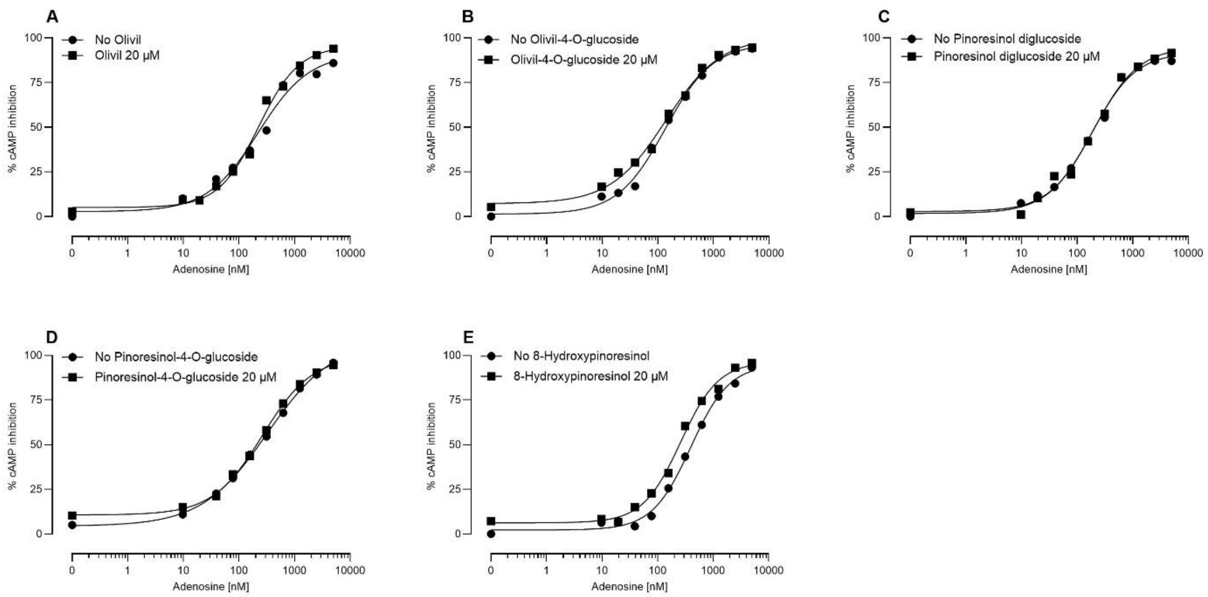

Figure S2. *Modulation of adenosine A1 receptor (A1AR) activity by lignans. The graphs illustrate the concentration-response curves for adenosine-induced cAMP inhibition in the presence and absence of various test compounds. Each test compound was evaluated at 20 µM to assess its potential allosteric modulation of A1AR signaling: (A) Olivil, (B) Olivil-4-O-glucoside, (C) Pinoresinol diglucoside, (D) Pinoresinol-4-O-glucoside, (E) 8-Hydroxypinoresinol. Data represent mean obtained from two experiments conducted in duplicate. Curves through the data represent the fit of an operational model of allosterism [28]. The presence of the test compounds resulted in no significant shift in the EC_{50} values or enhancement of maximal response, indicating that these lignans do not act as allosteric modulators of A1AR. The overlapping curves in each panel demonstrate that the test compounds neither enhanced nor inhibited the adenosine-mediated response.*

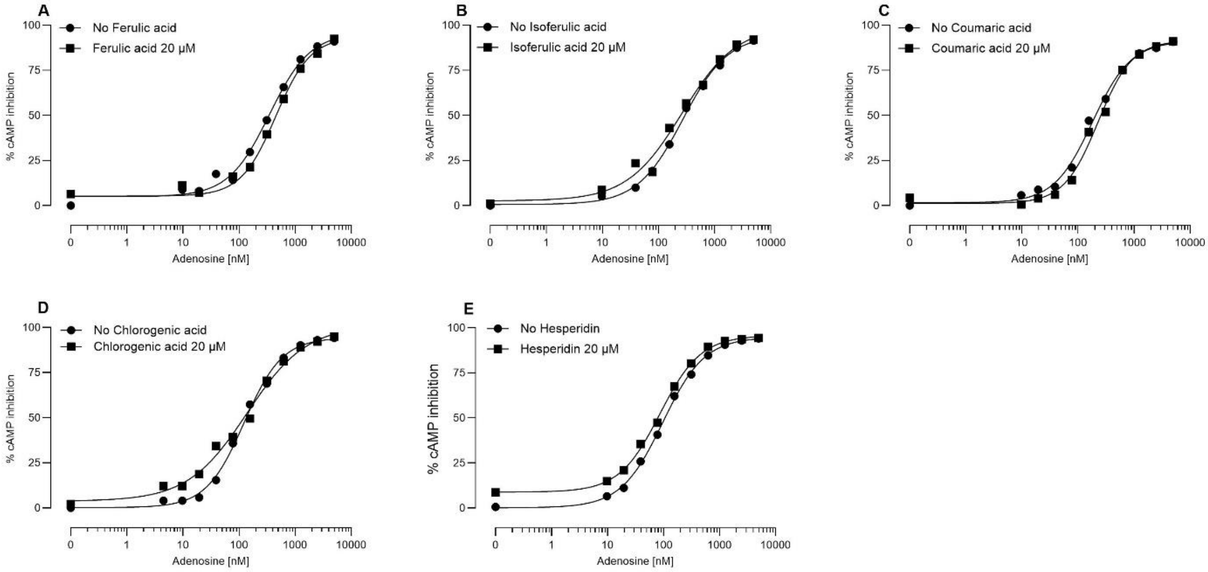

Figure S3. *Modulation of adenosine A1 receptor (A1AR) activity by caffeic acid derivatives and flavonoids. The graphs illustrate the concentration-response curves for adenosine-induced cAMP inhibition in the presence and absence of various test compounds. Each test compound was evaluated at 20 µM to assess its potential allosteric modulation of A1AR signaling: (A) Ferulic acid, (B) Isoferulic acid, (C) Coumaric acid, (D) Chlorogenic acid, (E) Hesperidin. Curves through the data represent the fit of an operational model of allosterism [28].The presence of the test compounds resulted in no significant shift in the EC_{50} values or enhancement of maximal response, except for Hesperidin, which caused a slight leftward shift in the dose-response curve, indicating potential partial allosteric modulation. The overall results suggest that most tested compounds do not exhibit allosteric modulation of the A1AR.*
